# Global Neuropeptide Annotations From the Genomes and Transcriptomes of Cubozoa, Scyphozoa, Staurozoa (Cnidaria: Medusozoa), and Octocorallia (Cnidaria: Anthozoa)

**DOI:** 10.3389/fendo.2019.00831

**Published:** 2019-12-06

**Authors:** Thomas L. Koch, Cornelis J. P. Grimmelikhuijzen

**Affiliations:** Section for Cell and Neurobiology, Department of Biology, University of Copenhagen, Copenhagen, Denmark

**Keywords:** neuropeptide, evolution, nervous system, Cnidaria, phylogeny

## Abstract

During animal evolution, ancestral Cnidaria and Bilateria diverged more than 600 million years ago. The nervous systems of extant cnidarians are strongly peptidergic. Neuropeptides have been isolated and sequenced from a few model cnidarians, but a global investigation of the presence of neuropeptides in all cnidarian classes has been lacking. Here, we have used a recently developed software program to annotate neuropeptides in the publicly available genomes and transcriptomes from members of the classes Cubozoa, Scyphozoa, and Staurozoa (which all belong to the subphylum Medusozoa) and contrasted these results with neuropeptides present in the subclass Octocorallia (belonging to the class Anthozoa). We found three to six neuropeptide preprohormone genes in members of the above-mentioned cnidarian classes or subclasses, each coding for several (up to thirty-two) similar or identical neuropeptide copies. Two of these neuropeptide preprohormone genes are present in all cnidarian classes/subclasses investigated, so they are good candidates for being among the first neuropeptide genes evolved in cnidarians. One of these primordial neuropeptide genes codes for neuropeptides having the C-terminal sequence GRFamide (pQGRFamide in Octocorallia; pQWLRGRFamide in Cubozoa and Scyphozoa; pQFLRGRFamide in Staurozoa). The other primordial neuropeptide gene codes for peptides having RPRSamide or closely resembling amino acid sequences. In addition to these two primordial neuropeptide sequences, cnidarians have their own class- or subclass-specific neuropeptides, which probably evolved to serve class/subclass-specific needs. When we carried out phylogenetic tree analyses of the GRFamide or RPRSamide preprohormones from cubozoans, scyphozoans, staurozoans, and octocorallia, we found that their phylogenetic relationships perfectly agreed with current models of the phylogeny of the studied cnidarian classes and subclasses. These results support the early origins of the GRFamide and RPRSamide preprohormone genes.

## Introduction

During animal evolution, ancestral Cnidaria, Placozoa, Ctenophora, and Porifera diverged from the Bilateria more than 600 million years ago (1). Neuropeptides have, so far, only been isolated and sequenced from cnidarians (2–4), although peptide-containing endocrine cells can also be found in placozoans and ctenophores (5, 6) and peptides have been annotated in placozoan genomes (7, 8). For understanding the origins and evolution of neuropeptides, therefore, it is important to study the four above-mentioned animal phyla with a focus, perhaps, on cnidarians, because they have well-developed peptidergic nervous systems (2–4).

**Figure 1:**
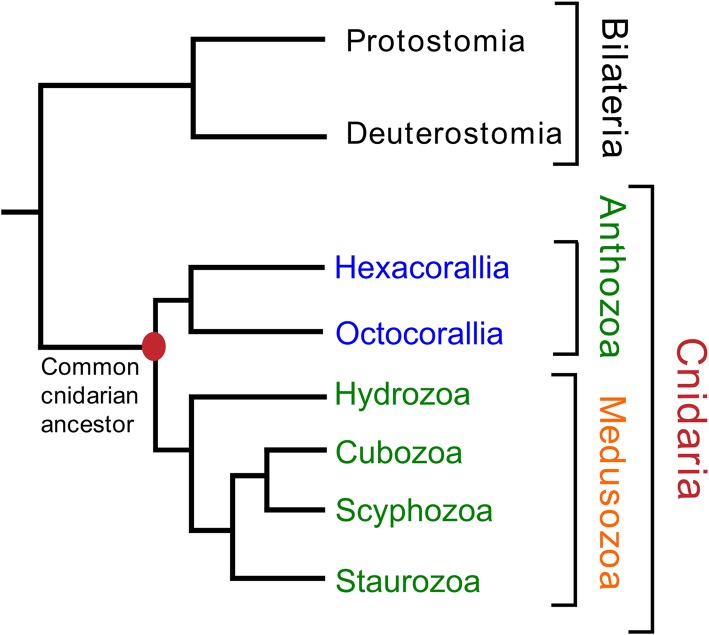
Phylogenetic relationships of the various cnidarian classes and subclasses. The class Myxozoa has been omitted in this figure, because of its uncertain position. Names highlighted in red indicate(s) a phylum (Cnidaria); in orange, a subphylum (Medusozoa); in green, a class; in blue, a subclass. The classes Staurozoa, Cubozoa, Scyphozoa, and Hydrozoa are often collected in the subphylum Medusozoa. The class Anthozoa can be subdivided into the subclasses Hexacorallia and Octocorallia. This figure is based on data from Technau and Steele (9), Zapata et al. (10), and Kayal et al. (11).

The phylum Cnidaria consists of six classes: Hydrozoa (*Hydra* and colonial polyps, such as *Clytia*), Scyphozoa (true jellyfishes), Cubozoa (box jellyfishes), Staurozoa (stalked jellyfishes), Anthozoa (sea anemones and corals), and Myxozoa (a group of small ectoparasites). Most Hydrozoa, Scyphozoa, Cubozoa, and Staurozoa have a life-cycle that includes a polyp and a medusa stage and these classes are, therefore, often collected into a subphylum named Medusozoa [see Figure 1, which is based on (9–11)]. The class Anthozoa is subdivided into two subclasses, Hexacorallia and Octocorallia, which have different rotational (radial) body symmetries, being a 6-fold rotational symmetry in Hexacorallia and an 8-fold rotational symmetry in Octocorallia.

Cnidarians have net-like nervous systems that sometimes are fused to form giant fibers or nerve rings in the bell margins of medusa, or are condensed in the head regions of polyps. These anatomical structures can be easily visualized in whole-mounts with the help of neuropeptide antibodies, because cnidarians are normally transparent (2, 12–14).

Cnidarian neuropeptides have only been isolated and sequenced from a few species such as the sea anemones *Anthopleura elegantissima*, and *Calliactis parasitica* (Hexacorallia), *Renilla Koellikeri* (Octocorallia), *Hydra magnipapillata* (Hydrozoa), and *Cyanea lamarckii* (Scyphozoa) (2–4, 15, 16). It was, therefore, unclear whether peptides occur ubiquitously in cnidarians and what the structures of these neuropeptides are.

In the last few years, several cnidarian genomes have been published (17–23) together with a large number of cnidarian transcriptomes (24–33). These important advancements in cnidarian biology open the possibility of tracking the evolution of the cnidarian neuropeptides and eventually determine the primordial neuropeptide(s) that evolved together with the early cnidarian nervous systems. In a recent paper we have developed a bioinformatics tool to predict neuropeptide preprohormone genes from several cubozoan transcriptomes (31). In our current paper we have applied this script to predict neuropeptide preprohormone genes in cnidarian species with publicly accessible genomes or transcriptomes that belong to three classes (Scyphozoa, Staurozoa, Cubozoa), all belonging to the Medusozoa. We have compared these data from Medusozoa with a prediction of neuropeptide genes present in Octocorallia, where this subclass was used as a kind of outgroup (see also Figure 1) to generate more contrasts in our results.

The aim of the current paper was to determine whether these cnidarian classes produce the same types of neuropeptides, or whether there exist class-specific neuropeptides. When common neuropeptides would be present in these classes, these peptides would be good candidates for being among the first neuropeptides that evolved during cnidarian evolution.

## Materials and Methods

### Sequence Data

We investigated assembled genomes (WGSs) and transcriptomes (TSAs) from seven octocorallians (*Renilla reniformis, Eleutherobia rubra, Xenia* sp. KK-2018, *Briareum asbestinum, Clavularia* sp., *Heliopora coerulera* and *Acanthogorgia aspera)*, three scyphozoans (*Aurelia aurita, Rhopilema esculentum*, and *Nemopilema nomurai)* and five staurozoans (*Calvadosia cruxmelitensis, Haliclystus aricula, Haliclystus sanjuanensis, Craterolophus convolvulus*, and *Lucernaria quadricornis*). The database accession numbers are shown in Table 1.

**Table 1:** Accession numbers for the different databases used.

**Species**	**Class**	**Database type**	**Accession number**
*Renilla reniformis*	Octocorallia	WGS	FXAL00000000.1
*Eleutherobia rubra*	Octocorallia	TSA	GHFI00000000.1
*Xenia* sp. *KK-2018*	Octocorallia	TSA	GHBC00000000.1
*Briareum asbestinum*	Octocorallia	TSA	GHBD00000000.1
*Clavularia* sp.	Octocorallia	TSA	GHAW00000000.1
*Heliopora coerulea*	Octocorallia	TSA	GFVH00000000.1 IABP00000000.1
*Acanthogorgia aspera*	Octocorallia	TSA	GETB00000000.1 GEXC00000000.1
*Rhopilema esculentum*	Scyphozoa	TSA	GEMS00000000.1
*Nemopilema nomurai*	Scyphozoa	TSA	GHAR00000000.1
*Aurelia aurita*	Scyphozoa	TSA	GBRG00000000.1
*Aurelia aurita*	Scyphozoa	WGS	REGM00000000.1
*Caladosia cruxmelitensis*	Staurozoa	TSA	HAHC00000000.1
*Calvadosia cruxmelitensis*	Staurozoa	WGS	OFHS00000000.1
*Haliclystus sanjuanensis*	Staurozoa	TSA	HAHB00000000.1
*Craterolophus convolvulus*	Staurozoa	TSA	HAGZ00000000.1
*Lucernaria quadricornis*	Staurozoa	TSA	HAHD00000000.1
*Haliclystus aricula*	Staurozoa	TSA	HAHA00000000.1

### Identification of Neuropeptide Preprohormones

We screened the translated genomes and transcriptomes for neuropeptide preprohormones using a script that is extensively explained in Nielsen et al. (31). This script is based on the presence of at least three similar peptide sequences followed by classical preprohormone processing sites (“GR” and “GK”) in the proteins. This script has, of course, its limitations, because neuropeptide genes might be missed that code for two or one neuropeptide copies on their preprohormones. Proteins with at least three processing sites were manually curated and labeled as neuropeptide preprohormones based on the presence of a signal peptide, the presence of three or more potential neuropeptide sequences, and the overall structure of the protein. The C-terminal parts of the immature neuropeptide sequences are easy to identify, because they are often followed by GR, GRR, or GKR sequences, which are classical processing sites for prohormone convertases (R, RR, KR), while the G residues are a classical processing signal for C-terminal amidation (3, 4). The N-termini, however, are often more difficult to determine as, in Cnidaria, N-terminal processing occurs by an unknown unspecific aminopeptidase cleaving at multiple residues, but stopping at Q residues, which are converted into N-terminal pQ groups (3, 4). In addition, N-terminal processing stops at N-terminal P or X-P sequences, which are also resistant to N-terminal degradation. The residues that are preferred for N-terminal processing are E, D, S, T, N, G, A, L, V, Y, or F (3). These residues often form the spacings in between the immature neuropeptide sequences on cnidarian preprohormones.

The identified neuropeptide preprohormones were also used as queries in TBLASTN searches against the other data sets using standard settings.

The putative preprohormones were investigated for the presence of signal peptides using SignalP 5.0 (http://www.cbs.dtu.dk/services/SignalP/) (34).

### Phylogenetic Analysis

The preprohormones were aligned using ClustalW (35). For phylogenetic tree analysis the aligned protein sequences were loaded in PAUP^*^^1^ and the maximum parsimony tree was calculated using p-distance and visualized in figtree ^2^.

## Results

### Annotation of Neuropeptide Preprohormones in Scyphozoa

Using our script for the discovery of neuropeptide preprohormones in cnidarian genomes and transcriptomes (31), we could detect six neuropeptide preprohormone genes in four publicly accessible databases from three scyphozoans: *Rhopilema esculentum* (transcriptome) *Nemopilema nomurai* (transcriptome), and *Aurelia aurita* (genome and transcriptome) (Table 1). The script detects neuropeptide genes that code for preprohomones that have three or more neuropeptide sequences, thus neuropeptide genes might be missed that code for two or one neuropeptide copies. Table 2 gives an overview of the neuropeptides contained in these six preprohormones. Supplementary Figures 1–6 give all the preprohormone sequences identified with our script in the three scyphozoan species.

**Table 2:**
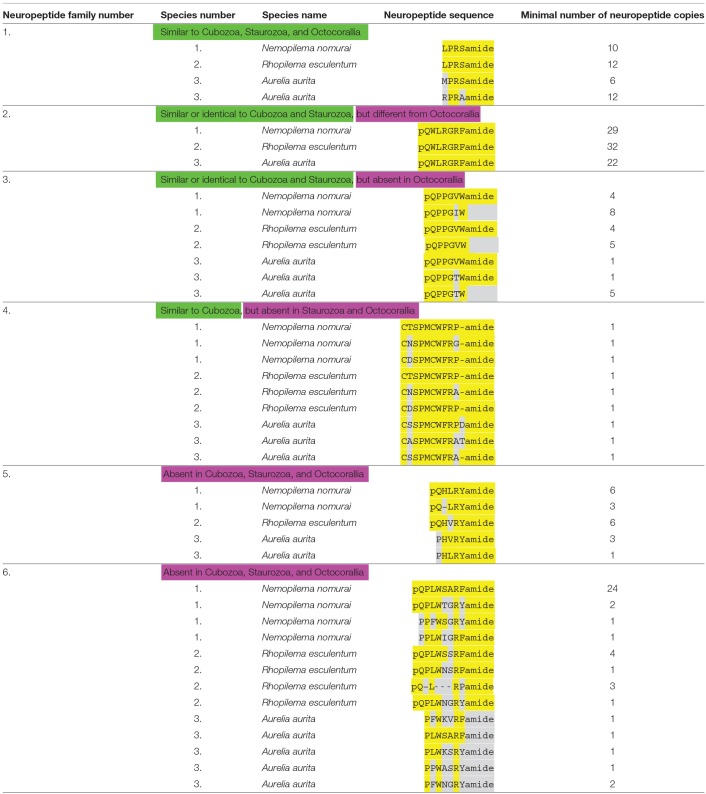
An overview of scyphozoan neuropeptide families.

*Only the major neuropeptides located on a preprohormone are listed here. The preprohormones are given in Supplementary Figures 1–6. Amino acid residues that are in common with the first-mentioned neuropeptide sequence from each neuropeptide family are highlighted in yellow*.

The scyphozoan databases from *N. nomurai, R. esculentum*, and *A. aurita* all contain transcripts and genes coding for a preprohormone that produce multiple copies of either LPRSamide or closely related neuropeptide sequences (Table 2; neuropeptide family #1). These sequences are flanked by classical GKR or GRR processing sites at their C-termini, where cleavage occurs C-terminally of K or R, after which the C-terminal G residues are converted into a C-terminal amide group (3, 4). At the N-termini of the neuropeptide sequences are acidic (E or D), or S, T, G, N, A, M, L, or V residues, which are processing sites that are often used in cnidarians for N-terminal neuropeptide processing (3, 4) (Supplementary Figure 1). In the proposed mature neuropeptide sequences, the C-termini are protected by an amide bond (for example LPRSamide), while the N-termini are protected by a prolyl residue in the second position of the peptide (Table 2). Similar LPRSamide neuropeptide sequences can also be detected in databases from Staurozoa (Table 3), Cubozoa (Table 4), and Octocorallia (Table 5).

**Table 3:**
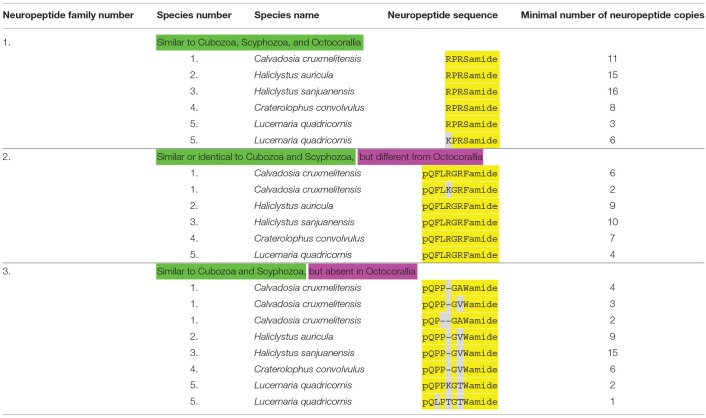
An overview of staurozoan neuropeptide families.

*Only the major neuropeptides located on a preprohormone are listed here. The preprohormones are given in Supplementary Figures 7–9. Amino acid residues are highlighted as in Table 2*.

**Table 4:**
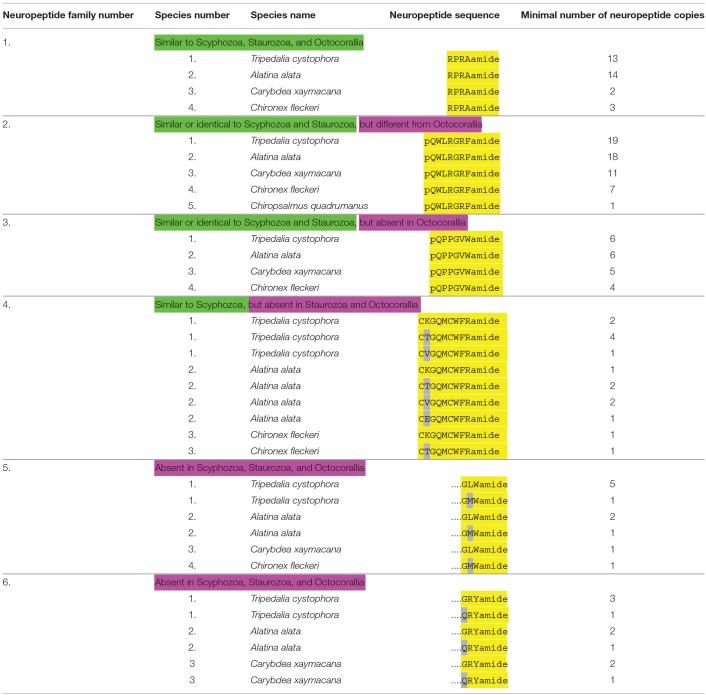
An overview of cubozoan neuropeptide families.

*This table is a shortened version of Table 1 from Nielsen et al. (31). Only the major neuropeptides are shown here. Amino acid residues are highlighted as in Table 2*.

**Table 5:**
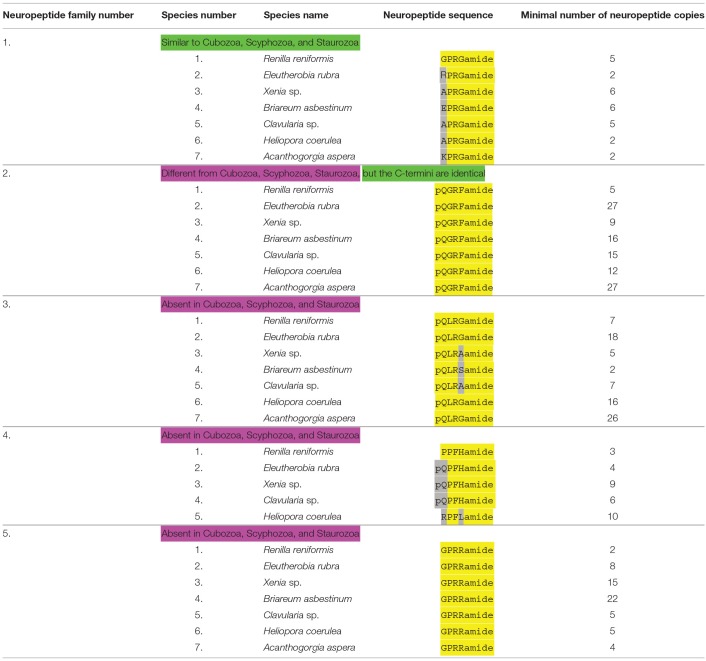
An overview of octocorallian neuropeptide families.

*Only the major neuropeptides located on a preprohormone are listed here. The preprohormones are given in Supplementary Figures 10–14. Amino acid residues are highlighted as in Table 2*.

The Scyphozoan databases from *N. nomurai, R. esculentum*, and *A. aurita* also contain transcripts and genes that code for numerous copies of identical pQWLRGRFamide neuropeptides (Table 2; neuropeptide family #2). These GRFamide neuropeptides have classical C-terminal GR or GKR processing sites and, again acidic (D or E), K, N, G, or S N-terminal processing sites that are often used in cnidarian N-terminal preprohormone processing (3, 4) (Supplementary Figure 2). Similar GRFamide peptides occur in Staurozoa (Table 3), and identical GRFamide neuropeptides occur in Cubozoa (Table 4). In Octocorallia GRFamide neuropeptides exist that have the C-terminal GRFamide sequence in common with the scyphozoan pQWLRGRFamide peptides (Table 5).

All four databases show that scyphozoans also produce a neuropeptide preprohormone that code for several copies of pQPPGVWamide and pQPPGTWamide and their non-amidated variants pQPPGVW and pQPPGTW (Table 2; neuropeptide family #3). These preprohormones have classical GKR, RR, RRR, RKR, or RKK processing sites at the C-termini of the neuropeptide sequences and N-terminal K, S, or N residues that in cnidarians are known to be involved in N-terminal processing (3, 4) (Supplementary Figure 3). Similar pQPPGVWamide peptides also occur in Staurozoa (Table 3; peptide family #3) and Cubozoa (Table 4; peptide family #3), but they are absent in Octocorallia (Table 5).

**Figure 2:**
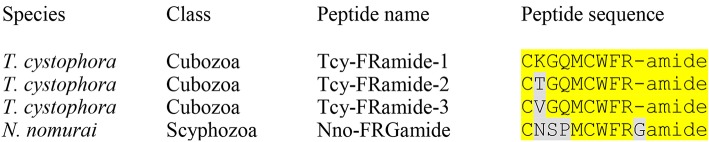
Alignment of the cyclic FRamide peptides from the cubozoan *T. cystophora* and the scyphozoan *N. nomurai*. Amino acid residues that are in common with the cubozoan sequence CKGQMCWFRamide, named Tcy-FRamide [see Table 1 from Nielsen et al. (31)] are highlighted in yellow. Please note that the peptides are cyclic after the cysteine residues have formed a cystine bridge and that the resulting “loops” are six-rings with the same size in all peptides.

The *N. nomurai* transcriptome database also codes for a preprohormone that contains one copy of a probable cyclic neuroepeptide CTSPMCWFRPamide and several other nearly identical peptides, where the two cysteine residues are likely forming a cystine bridge (Table 2; neuropeptide family #4; Supplementary Figure 4). Similar peptides can be found in the databases from *R. esculentum*, where a small number of amino acid residue exchanges occur without, however, changing the consensus sequence CXSPMCWFRXamide (Table 2; peptide family #4). In *A. aurita*, however, the C-termini are extended by one or two amino acid residues (Table 2; peptide family #4). The C-termini of all neuropeptide sequences in the preprohormones are followed by classical GR, GKR, GKKR, or GKRR processing sites and the neuropeptide sequences are preceded by G, N, D, E, or S sequences, which are known processing sites in cnidarian preprohormones (3, 4) (Supplementary Figure 4). Similar cyclic neuropeptides can also be found in the transcriptomes of three cubozoans, which all have the consensus sequence CXGQMCWFRamide (Table 4, Figure 2). Thus, compared to the scyphozoan neuropeptides, these cubozoan neuropeptides have the sequences CXXXMCWFRamide in common, including a common distance between the cysteine residues forming the presumed cystine bridge (Figure 2). These neuropeptides could not be found in Staurozoa (Table 3), or Octocorallia (Table 5).

Finally, Scyphozoans have two neuropeptide families that cannot be found in Staurozoa, Cubozoa, and Octocorallia and which, therefore, appear to be scyphozoan-specific (Table 2; peptide families #5 and #6).

In *N. nomurai*, the first neuropeptide family (neuropeptide family #5 of Table 2; Supplementary Figure 5) consists of members having the sequence pQHLRYamide or other very similar sequences. In *R. esculentum*, six copies of the sequence pQHVRYamide can be identified (Table 2). In *A. aurita*, a prolyl residue, which also protects the N-terminus of a neuropeptide, is replacing the pyroglutamyl residue: PHVRYamide and PHLRYamide (Table 2). In the preprohormones for these neuropeptides the neuropeptide sequences have classical GR or GK processing sites at their C-termini, while at their N-termini they have R, D, T, and A residues, which in cnidarians are known to be involved in N-terminal processing (3, 4) (Supplementary Figure 5).

The second scyphozoan-specific neuropeptide family (neuropeptide family #6 of Table 2; Supplementary Figure 6) consists of pQPLWSARFamide or related sequences in *N. nomurai* (Table 2). For some peptides the N-terminal pyroglutamyl group is lacking, but those peptides still have two sequential N-terminal prolyl residues, which protect the N-termini against enzymatic degradation (Table 2). *R. esculentum* have peptides that are very similar to the ones occurring in *N. nomurai* with the exception of three copies of a short peptide pQLRPamide that do not occur in *N. nomurai*. All peptides from *A. aurita* lack N-terminal pyroglutamyl residues, but, again, are N-terminally protected by prolyl residues (Table 2).

### Annotation of Neuropeptide Preprohormones in Staurozoa

Using our script (31), we discovered three different neuropeptide preprohormone genes in staurozoans (Table 3). In *Calvadosia cruxmelitensis* we found a preprohormone that contained 11 copies of the neuropeptide sequence RPRSamide (Table 3, neuropeptide family #1; Supplementary Figure 7). These RPRSamide sequences have the classical C-terminal processing site GKR, while N-terminally of the RPRSamide sequences are D, V, I, F, and A residues, which in cnidarians are known preprohormone processing sites (3, 4). Similar preprohormones exist in the transcriptomes from *Haliclystus auricula* and *Haliclystus sanjuanensis* (Supplementary Figure 7), which contain 15, respectively, 16 copies of the RPRSamide sequence (Table 3). In the *Craterolophus convolvulus* transcriptome we could identify a preprohormone with 8 copies of the RPRSamide sequence. In the transcriptome from *Lucernaria quadricornis* we discovered a neuropeptide preprohormone with 3 copies of the RPRSamide and 6 copies of the KPRSamide sequence (Table 3; Supplementary Figure 7). As mentioned earlier, neuropeptide preprohormones having numerous copies of RPRSamide or similar peptides, also occur in Scyphozoa (Table 2), Cubozoa (Table 4), and Octocorallia (Table 5).

The transcriptomes from the four Staurozoa species also contain transcripts coding for GRFamide preprohormones (Table 3, neuropeptide family #2). For all species these preprohormones contain numerous copies of the neuropeptide pQFLRGRFamide (Table 3; Supplementary Figure 8). This neuropeptide is very similar to the one from scyphozoans (Table 2, neuropeptide family #2), but it contains an F residue at position 2 instead of a W residue in the scyphozoan peptide. The same is true for cubozoans (Table 4, neuropeptide family #2), where the GRFamide peptides also have a W residue at position 2. However, compared to Octocorallia (Table 5, neuropeptide family #2) the staurozoan GRFamide peptides are much longer, being N-terminally elongated by three amino acid residues.

The third neuropeptide preprohormone that we discovered in staurozoans contains numerous copies of a pQPPGAWamide neuropeoptide or closely related sequences (Table 3, neuropeptide family #3; Supplementary Figure 9). All peptides are protected by amino-terminal pQ, pQP, or pQPP sequences against unspecific aminoterminal enzymatic degradation. Identical or similar neuropeptides occur in scyphozoans (Table 2, neuropeptide family #3) or cubozoans (Table 4, neuropeptide family #3). The peptides, however, are absent in Octocorallia (Table 5).

### Annotation of Neuropeptide Preprohormones in Cubozoa

We have recently annotated neuropeptide preprohormones in five different cubozoan species (31). A short summary of these results is given in Table 4, while the amino acid sequences of the preprohormones are given in Nielsen et al. (31).

Cubozoans produce RPRAamide neuropeptides (Table 4, neuropeptide family #1) that are very similar to neuropeptides occurring in scyphozoans (Table 1, neuropeptide family #1), staurozoans (Table 3, neuropeptide family #1) or octocorallians (Table 5, neuropeptide family #1).

Cubozoans also have preprohormones that produce numerous copies of pQWLRGRFamide (Table 4, neuropeptide family #2). Identical or very similar neuropeptides can also be found in scyphozoans (Table 2, neuropeptide family #2), and staurozoans (Table 3, neuropeptide family #2). However, in octocorallians there is a shorter version of these peptides (Table 5, neuropeptide family #2).

Cubozoans produce the neuropeptide pQPPGVWamide (Table 4, neuropeptide family #3) that is identical or very similar to neuropeptides produced in scyphozoans (Table 2, neuropeptide family #3), and staurozoans (Table 3, neuropeptide family #3). These peptides, however, do not occur in octocorallians (Table 5).

Cubozoans have peptides with the sequence CXGQMCWFRamide, which are probably cyclic after the two cysteine residues have formed a cystine bridge (Table 4, neuropeptide family #4). Scyphozoans have similar peptides (Table 2, neuropeptide family #4). However, these peptides are lacking in Staurozoa (Table 3) and Octocorallia (Table 5).

Finally, cubozoans have two peptide families that do not occur in Scypho- and Staurozoa. These are neuropeptides with the C-terminal sequence GLWamide (Table 4, neuropeptide family #5), and neuropeptides with the C-terminal sequence GRYamide (Table 4, neuropeptide family #6). The C-termini from the GLWamides are relatively well-conserved, but their N-termini are quite variable [see Table 1 from Nielsen et al. (31)]. The same is true for the GRYamide peptides (31).

### Annotation of Neuropeptide Preprohormones From Octocorallia

We investigated the transcriptomes from seven Octocorallia species for the presence of neuropeptide genes (Tables 1, 5).

Octocorallia produce GPRGamide and closely related peptides (Table 5, neuropeptide family #1; Supplementary Figure 10) that resemble very much the LPRSamide and RPRAamide peptides from scyphozoans (Table 2, neuropeptide family #1), staurozoans (Table 2, neuropeptide family #1), and cubozoans (Table 4, neuropeptide family #1).

All octocorallians produce a preprohormone that carry numerous copies of the neuropeptide pQGRFamide (Table 5, neuropeptide family #2; Supplementary Figure 11). This peptide, dubbed Antho-RFamide, was the first cnidarian neuropeptide to be chemically isolated and sequenced from Anthozoa (36), including the octocoral *Renilla* (37). The presence of acidic residues, preceding the peptide sequence in the *Renilla* preprohormone (Supplementary Figure 11) illustrates, again, that processing must occur at E or D residues. Antho-RFamide does not occur in Scyphozoa, Staurozoa, and Cubozoa, but these medusazoans have N-terminally elongated forms that have the C-terminal GRFamide sequence in common with Antho-RFamide (neuropeptide families #2 from Tables 2–4). Thus, GRFamide neuropeptides are widespread in cnidarians.

In contrast to the two neuropeptide families discussed above that appear to be ubiquitous in cnidarians, octocorallians also produce Octocorallia-specific neuropeptides. The first group (Table 5, neuropeptide family #3) has the structure pQLRGamide or a very similar sequence. Their preprohormones are given in Supplementary Figure 12.

The second group of neuropeptides (Table 5, neuropeptide family #4) has the structure PPFHamide, or pQPFHamide. Both sequences are N-terminally protected by either the N-terminal PP or pQP sequences. Their preprohormones are given in Supplementary Figure 13. In addition, we discovered a preprohormone in *H. coerulea* that produces multiple copies of an RPFLamide sequence. Also these peptides are N-terminally protected by prolyl residues at position 2, but they are only 50% identical with the other peptides from this family (Table 5, peptide family #4; Supplementary Figure 13).

The third group of Octocorallia-specific neuropeptides (Table 5, neuropeptide family #5; Supplementary Figure 14) has the sequence GPRRamide, or a closely related sequence. Again, these neuropeptide sequences are protected against N-terminal enzymatic degradation by a prolyl residue at position 2 of the peptides. The *R. reniformis* preprohormone has at least two copies of GPRRamide, the *E. rubra* preprohormone produces eight copies of GPRRamide, the *Xenia* sp. preprohormone contains 15 GPRRamide copies, the *B. asbestinum* preprohormone 22 copies, the *clavularia* sp. preprohormone 5 copies, and the *H. coerulea* preprohormone 4 copies of GPRRamide (Table 5, neuropeptide family #5; Supplementary Figure 14).

### Phylogenetic Tree Analyses of the XPRXamide and GRFamide Preprohormones

**Figure 3:**
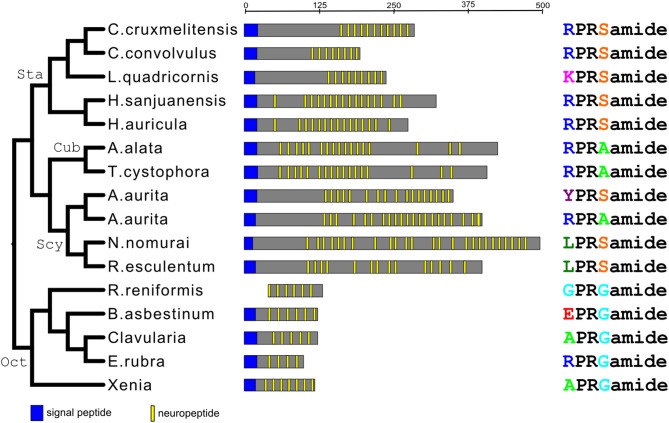
Phylogenic tree analysis of all the identified X_1_PRX_2_amide preprohormones from scyphozoans, staurozoans, cubozoans, and octocorallians. This phylogenetic tree (left panel) is in complete accordance with the phylogenetic tree given in Figure 1. The abscissa (upper line) gives the length of the preprohormone fragments (in amino acid residues). The middle panel gives schematic representations of the various preprohormones from each species. The right panel gives the amino acid sequences of the major X_1_PRX_2_amide peptides produced by these preprohormones. This panel also shows that X_1_PRX_2_amide (where X_2_ is either S, A, or G) is the consensus sequence of all peptides.

We carried out a phylogenetic tree analysis of all the XPRSamide/XPRAamide/XPRGamide preprohormones investigated in this paper [neuropeptide families #1, from Tables 2–5; Supplementary Figures 1, 7, 10; (31)]. These studies (Figure 3) showed that the structural relationships between these preprohormones very precisely followed the established phylogenetic relationships of the classes and subclasses they belong to (Figure 1). These findings show that all XPRSamide/XPRAamide/XPRGamide preprohormones are derived from a common ancestor.

**Figure 4:**
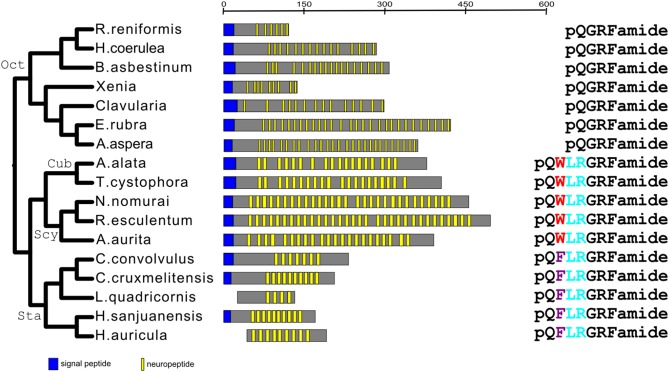
Phylogentic tree analysis of all the identified GRFamide preprohormones from scyphozoans, staurozoans, cubozoans, and octocorallians. Like in Figure 3, also this phylogenetic tree is in complete agreement with the phylogenetic tree given in Figure 1. The panels are organized as in Figure 3. The right panel gives the peptide sequences. This panel shows that all the Octocorallia species produce identical peptides, which is in agreement with their close phylogenetic relationship. Similarly, this panel shows that the cubozoans and scyphozoans produce identical peptides suggesting that these classes are more related to each other than to the staurozoans, which produce a slightly different peptide.

When we carried out the same analysis for the GRFamide preprohormones, we came to the same conclusion (Figures 1, 4). Aligning the mature neuropeptide sequences themselves (right panel of Figure 4), showed that the octocorallian peptide pQGRFamide is farthest away from the other GRFamide peptides, while the pQWLRGRFamides are identical in both Cubo- and Scyphozoa and only slightly different from the pQFLRGRFamides that occur in Staurozoa. These findings are, again in complete agreement with the phylogenetic relationships between the classes and subclasses to which these peptides belong (Figures 1, 4), showing that all GRFamide preprohormones are derived from a common ancestor.

## Discussion

In our paper we have analyzed the neuropeptide preprohormones from three cnidarian classes (Scyphozoa, Cubozoa, Staurozoa) and one subclass (Octocorallia). We did not include the remaining classes (Hydrozoa and Myxozoa) and subclasses (Hexacorallia) in our study, because already the current study includes a large amount of data (Supplementary Figures 1–14) with altogether 66 preprohormones, each of which contains a varying number of different neuropeptides (Tables 2–5). These large numbers of preprohormones and neuropeptides are difficult to analyze and present in an understandable and concise way. Yet, although not all cnidarian classes have been included in our analyses, we can already now draw conclusions related to the major questions that we asked at the end of the Introduction: (i) Do all cnidarian classes produce the same types of neuropeptides; or (ii) are there class-specific neuropeptides?

**Figure 5:**
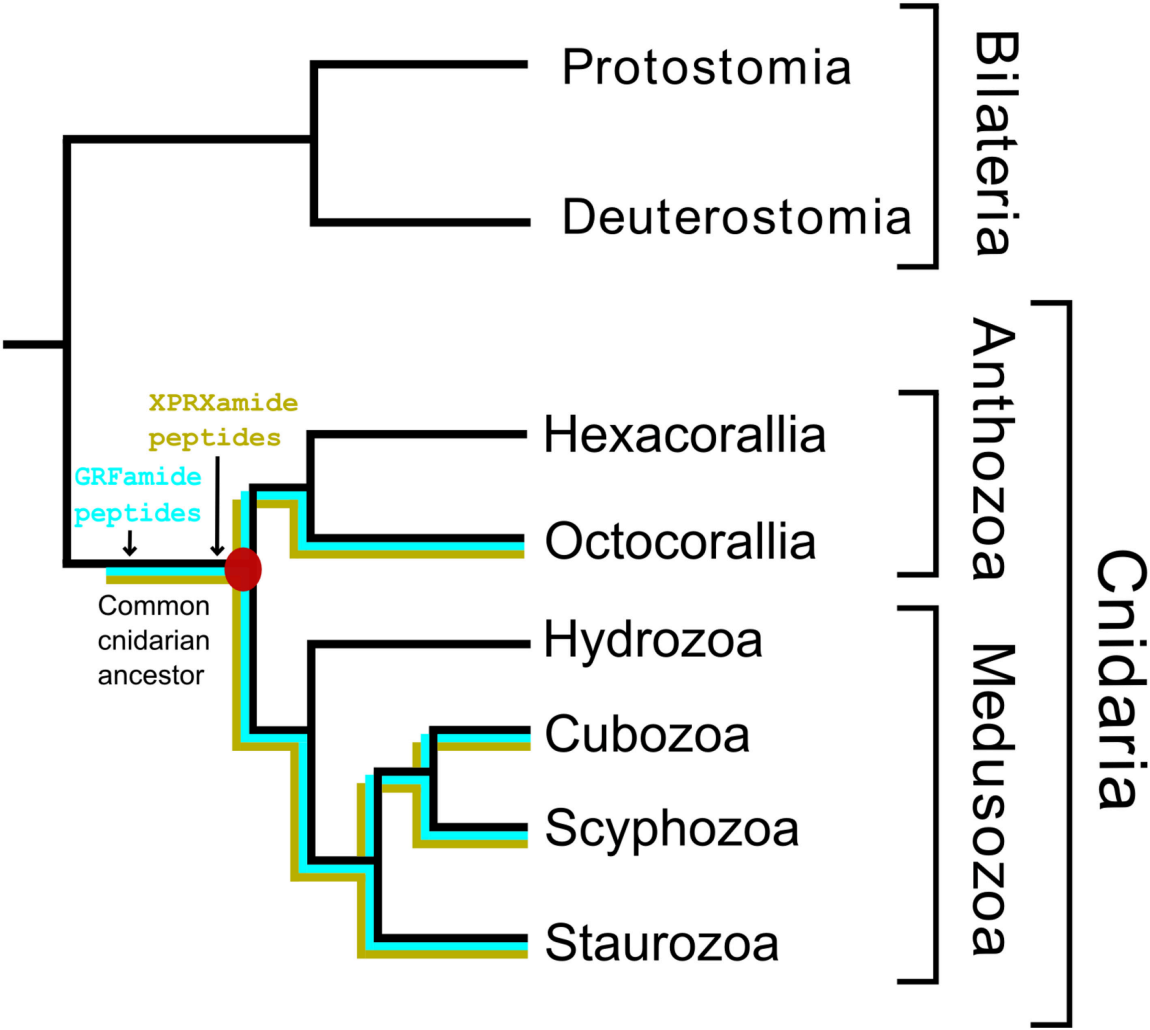
A schematic drawing illustrating the evolution of some neuropeptides in Cnidaria. This figure has the same structure as Figure 1. It shows that the GRFamide peptides (highlighted in blue) and the X_1_PRX_2_amide peptides (highlighted in yellow) originated before or in the common cnidarian ancestor (indicated by a filled red circle).

We found that Scyphozoa, Cubozoa, Staurozoa, and Octocorallia all produced GRFamide peptides (Figure 4), which is an answer to the above-mentioned question (i). This finding also means that the common cnidarian ancestor (red filled circle in Figure 5) must have produced GRFamides, suggesting that GRFamides are ancient neuropeptides that probably evolved together with the first cnidarians. pQGRFamide (Antho-RFamide) is a well-established neuropeptide that has been isolated, sequenced and cloned from the sea anemones *Anthopleura elegantissima* and *Calliactis parasitica* (Hexacorallia) and from the sea pansy *Renilla koellikeri* (Octocorallia) (3, 4, 36–39). Using immunocytochemistry, dense nets of Antho-RFamide producing neurons have been found in *C. parasitica* and *R. koellikeri* (2, 40), showing that these peptides are genuine neuropeptides.

N-terminally elongated forms of Antho-RFamide have been isolated and sequenced from Scyphozoa, such as pQWLRGRFamide from *Cyanea larmarckii* (compare right panel of Figure 4; and Table 2, peptide family #2) and the use of antibodies showed its presence in nerve nets of *C. lamarckii* (41, 42). Thus, both Antho-RFamide and its N-terminally elongated forms (right panel of Figure 4) are well-established neuropeptides in Cnidaria.

There is another peptide-family that occurs in all Octocorallia, Cubozoa, Scyphozoa, and Staurozoa species that we have investigated. These peptides have the general structure X_1_PRX_2_amide, where X_1_ is quite variable, while X_2_ is confined to S, A, or G (neuropeptide family #1 from Tables 2–5; right panel of Figure 3). Because these peptides occur in both Octocorallia and the three classes that belong to the Medusozoa (Figure 5), it is likely that they also were present in the common cnidarian ancestor (red filled circle in Figure 5).

After submission of our manuscript and two reviewing rounds, a paper was published on neuropeptides present in the hexacoral *N. vectensis* (43). This paper confirms the presence of GRFamide and X_1_PRX_2_ peptides in Hexacorallia, which supports our conclusion that these two neuropeptide families originated in the common cnidarian ancestor (Figure 5). Furthermore, this paper confirms the presence of class-specific neuropeptides (see below).

In contrast to the GRFamides, not much is known about the XPRXamides. However, two peptides related to this peptide family (WPRPamide and RPRPamide) have recently been identified in the hydrozoan jellyfishes *Clytia hemispherica* and *Cladonema pacificum* as the endogenous neuropeptides inducing oocyte maturation, and oocyte and sperm release (44). The peptides have also been localized in neurons and, therefore, are genuine neuropeptides (44).

If one looks at the structures of the various X_1_PRX_2_amide peptides (right panel of Figure 3), one can see several interesting features. First, all peptides have a prolyl residue at position 2. This residue might help protecting the neuropeptide against non-specific enzymatic N-terminal degradation, because the X-P bond is not an amide, but an imide bond, which likely gives resistance against enzymatic hydrolysis. Prolyl residues have also been found at the N-termini of many other peptides described in this paper (for example Table 2, peptide family #6; Table 5, peptide families #4 and #5). Second, none of the X_1_PRX_2_amide peptides are protected by an N-terminal pQ group, while most of the other neuropeptide families (Tables 2–5) have such protecting groups, suggesting that the X_1_PRX_2_amide peptides need an N-terminal positive charge (by the protonation of the N-terminal primary amine group) for binding to their G-protein-coupled receptor (GPCRs). Third, the R residue at position 3 of the peptide is conserved in all peptides, creating, again, a positive charge in the middle of the peptide and making the overall charge of all peptides in the family quite positive, especially when the residues in position 1 are R or K (Figure 3, right panel).

In Octocorallia there is, in addition to the X_1_PRX_2_amide peptides (where X_2_ is S, A, or G), another peptide family, of which most members have the GPRRamide sequence (Table 5, neuropeptide family #5). However, we do not think that these peptides belong to the same family as the X_1_PRX_2_amide family, because their preprohormones have a different organization. While in many X_1_PRX_2_amide preprohormones, most neuropeptide sequences are followed by a dibasic (KR) processing site [Supplementary Figures 1, 7, 10; (31)], there are exclusively single basic (R) processing sites at these positions in the GPRRamide precursor (Supplementary Figure 14). Furthermore, in all GPRRamide preprohormones, the neuropeptide sequence is frequently preceded by the sequence DEIT (Supplementary Figure 14), whereas this sequence is absent in all X_1_PRX_2_ preprohormones [Supplementary Figures 1, 7, 12; (31)]. We assume, therefore, that the GPRRamide preprohormones might not be evolutionarily closely related to the X_1_PRX_2_amide preprohormones.

Besides the GRFamides and X_1_PRX_2_amides all other peptide families described in this paper are novel, with exception of the peptides given in Table 4, which have been published earlier (31), and we do not really know whether they are localized in neurons and, thus, are genuine neuropeptides. These peptides are not ubiquitous in cnidarians, but often occur in more than one class. For example, the presumed cyclic peptides from scyphozoans (Table 2, neuropeptide family #4) do also occur in cubozoans (Table 4, neuropeptide family #4). These results establish the close relationships between scyphozoans and cubozoans, which again is in accordance with current models of the phylogeny of cnidarian classes (Figure 1).

Also the pQPPGVWamide peptide family (Table 2, neuropeptide family #3; Table 3, neuropeptide family #3; Table 4, neuropeptide family #3) occurs in scypho-, cubo-, and staurozoans. These results confirm the close phylogenetic relationships between these classes, which is in full agreement with the current models for cnidarian phylogeny (Figure 1).

In addition to these peptide families that occur in more than one classes, there are neuropeptides that are confined to a single class (Table 2, neuropeptide families #5 and #6; Table 4, neuropeptide families #5 and #6; Table 5, neuropeptide families #3, #4, #5). These neuropeptides may serve class-specific physiological processes.

None of the above-mentioned peptides identified in the four cnidarian classes/subclasses have significant structural similarities with any of the known bilaterian neuropeptides.

## Data Availability Statement

The datasets for *T. cystophora* can be found in the GenBank GGWE01000000.

## Author's Note

This paper is part of the article collection “The Evolution of Neuropeptides: A Stroll through the Animal Kingdom: Updates from the Ottawa 2019 ICCPB Symposium and Beyond” hosted by Dr. Klaus H. Hoffmann and Dr. Elisabeth Amy Williams.

## Author Contributions

TK and CG conceived and designed the project, and analyzed the data. TK carried out the experiments. CG wrote the paper with inputs from TK. All authors approved the final manuscript.

### Conflict of Interest

The authors declare that the research was conducted in the absence of any commercial or financial relationships that could be construed as a potential conflict of interest.

## Footnotes

^1^https://paup.phylosolutions.com/ (accessed September 20, 2019).

^2^https://github.com/rambaut/figtree (accessed September 20, 2019).
