# Supplementary material for: Global Neuropeptide Annotations From the Genomes and Transcriptomes of Cubozoa, Scyphozoa, Staurozoa (Cnidaria: Medusozoa), and Octocorallia (Cnidaria: Anthozoa)
Source: Front Endocrinol (Lausanne). 2019 Dec 6;10:831. doi: 10.3389/fendo.2019.00831 (PMC6909153; doi:10.3389/fendo.2019.00831)
Supplement: Supplementary file 1 [file Data_Sheet_1.PDF]

Supplementary Fig. 1. Partial amino acid sequences of the LPRSamide preprohormones in scyphozoans. Signal sequences are underlined. Neuropeptide sequences are highlighted in yellow; C-terminal processing sites are highlighted in green. The C-terminal Gly residues that are converted into C-terminal amide groups are highlighted in red.

Nemopilema nomurai

> Nemopilema nomurai isolate NNO-Tongyong01 scaffold106_contig1, whole genome shotgun sequence

MELLWVLQLFIACYAQYVCCGRTATVAEDLALLSDQVTSQQEESTSELLLDLVKQLEKYCGTSSNLDQNVCRN
ALHYLLSNFENFDSLMEEEKKGLDGAVSEDSLPRSGKREVEEEEMLEGMLPRSGKREMDLPRSGKRMLEMEKE
LPRHGKRDFVLPRSGKREMDLPRSGKRMLEMEEELPRHGKRNFRLPRSENREVDMLEVDEIMGRQEEERDVYL
PRSGKRGHMVRLLNQQDRSRFEDLAMPRYGKKELPRAGRRVAARPGRREMSLPRSGKREINLPRSGKRSLKRS
EAREENESEKDLAEMSESTKRSIALPRSGREFSMLPRSGKRASATQGELVARRESYFPRSGREQTGQAEESFE
LSREMGMLRPGKRNTARMLRPGKRNSVEALRPGKRNSVEALRPGKRNTVEALRPGKRNTVEALRPGKRDSFEI
LRPGKRNTVEALRPGKRNSVVILRPGKRNSVEILRPGKREEVIETETEDELRSKAFATS

Rhopilema esculentum

>GEMS01057357.1 TSA: Rhopilema esculentum c62341_g1_i1 transcribed RNA sequence

MKLILVSLLSFSLYAQFVSCRRKISVAEDLALLNDQIISQQQKSTSELLLELVKQLEKYCGTSSDLDRNVCRN
ALHYLLSNFDNFDSSMQEEKKNARFSAVASEDALPRSGKREEVSLPRSGKRENIDLPRSGKRELALPRSGKRL
VELRRKIQRYGKRESRLARSGKREVDMFEVGEKKRNVHLPRSGKREMETRASGRQEISRYRKRNFAMPRYGRR
ELELPRAGKRKTEMLWFEKRERRLPRSGKREFDLPRSGKRESAMARSVTEGERSAEDVTERSSEKTVASFVKV
DDESMKRSMTLPRSGREYIMLPRSGKRAFVASERELPRSGKRRAVLAAGEENYLPRSGREEAMHAKRNLELSG
ELELPRSGKRQLVEGKQVDGADPEEELEDNAFMAS

Aurelia aurita

>GBRG01078128.1:435-1487 TSA: Aurelia aurita comp187729_c0_seq1 transcribed RNA sequence

MRALKIMLAVVALVFLSVQYAYSLNAELLADELAMVDAEETGQAYKSTSQLLLKLVEQLEKYCGTSSESDRDL
CQNALRYLLGNFNDFDELMNEANAGGKDQARNIINKEGRVRSESGDDEAASMVLVSEREHMGMPRSGKRESEY
PRSGKRDTEMPRSGKRGIEMSRSGKRDFEMPRSGKRGSDKMAENRELGTSILAEDRSGYPRSGKRELELATAR
DRIGMPRSGKREADDYPRSGKREADVTQSEMQMNDRLRAGRNMQRSGKREADYPRSSKREADYPRSGKREADY
PRSGKREADYPRSGKRDAEMPRSGKRNIEMPRSGKRGMEMPRSGKRGIDMPRYGKRGIE

>REGM01000384.1:126762-127760 Aurelia aurita isolate ABS-J01 scaffold384, whole genome shotgun sequence

MEAMKIMLAVVVLACLSVQCADSLNAELLADELEVVDAEETNQGYKSTSELLLTLVEQLEKYCGTSSESERNL
CQNALRYLLGNFNDFDELMKEANAGGKDRASAIISEGGQGERGDEELASMLLVSERERMGMPRSGKRENKYPR
SGKRDIALPRSGKRESDEMEEKRESDDLLFAEDISGYPRSGKKELKLAAAMDRMSMPRSGRRAADDYPRSGRG
EVDMMNSAMQMNDRPRAGKRVVERPRAGKREFERPRAGKRELDRPRAGKREIDRPRAGKRDLGRPRAGKRELD

RPRAGKREINRPRAGKRELDLERPRAGKRELDRPRAGKRELDRPRAGKRELDIERPRAGKREFEILNTGSEEN
DFPRSGKRDIDERDIETPRSGKRELDYPRSGTGRK
