# Supplementary material for: Global Neuropeptide Annotations From the Genomes and Transcriptomes of Cubozoa, Scyphozoa, Staurozoa (Cnidaria: Medusozoa), and Octocorallia (Cnidaria: Anthozoa)
Source: Front Endocrinol (Lausanne). 2019 Dec 6;10:831. doi: 10.3389/fendo.2019.00831 (PMC6909153; doi:10.3389/fendo.2019.00831)
Supplement: Supplementary file 2 [file Data_Sheet_2.PDF]

Supplementary Fig. 2. Partial or complete amino acid sequences of the GRFamide preprohormones in scyphozoans. The sequences are highlighted as in Supplementary Fig. 1.

Nemopilema nomurai

>gi|1529711544|gb|PEDN01001281.1|_selection_translation_frame_+3

MAKIINVALCALLISQVLCEVIETKKTKDPDEEEDPEQLEDAKRSIKQWLRGRFGKREDNQWLRGRFGKREDE
QWLRGRFGKREDEQWLRGRFGKREDEQWLRGRFGKREDGQWLRGRFGKREDEQWLRGRFGKREDGQWLRGRFG
KREDGQWLRGRFGKREDEQWLRGRFGREGSQWLRGRFGKEAEQWLRGRFGREAGQWLRGRFGREAGQWLRGRF
GKEAGQWLRGRFGKEAEDEEEGLEDEFEQWLRGRFGKEVDEQWLRGRFGREALQWLRGRFGRDLAEQWLRGRF
GKEAEEQWLRGRFGKETDEEDQWLRGRFGKEAAEQWLRGRFGKEAEDQWLRGRFGKEAEEADQWLRGRFGKEA
EEQWLRGRFGREAFEQWLRGRFGRDADEQWLRGRFGKEAEQWLRGRFGREGEQWLRGRFGRDNTSSSDEKDAK
KDEAKANEKKGIEKAEKSKK

Rhopilema esculentum

> Rhopilema esculentum Unigene0032526 transcribed RNA sequence

MAKILSTVLCALLIYQVLCEKIEKKETKDNEKVGDKEQLDDAKRSIKQWLRGRFGKREDEQWLRGRFGKREDE
QWLRGRFGKREDEQWLRGRFGREDEQWLRGRFGKREDEQWLRGRFGKREDEQWLRGRFGKREDEQWLRGRFGR
EDEQWLRGRFGKREDEQWLRGRFGKREDEQWLRGRFGKREDEQWLRGRFGKREDEQWLRGRFGKREGEQWLRG
RFGKREDEQWLRGRFGRGAEQWLRGRFGREAGQWLRGRFGREAEQWLRGRFGKEAEDEDDGLEDEFEQWLRGR
FGKEVDEQWLRGRFGREALQWLRGRFGRELAEQWLRGRFGKEAEEQWLRGRFGKETDEADQWLRGRFGKEAEE
QWLRGRFGKEAAEQWLRGRFGKEAEEQWLRGRFGKEAEEQWLRGRFGRDALEQWLRGRFGRDSEEQWLRGRFG
KEAEQWLRGRFGRDFADQWLRGRFGRGSDSSNDEKDAKTDVAKESERKGGEKDLGSADKSK

Aurelia aurita

>GBRG01115465.1:296-1477 TSA: Aurelia aurita comp193240_c0_seq2
transcribed RNA sequence

MNLTLQIVLCAVFMTYALCESTEKKETPGAQSENEKLGPAKRALEQWLRGRFGRESGKEPRELDQWLRGRFGK
REDSQWLRGRFGREAKQWLRGRFGKEADENEEALESEFEQWLRGRFGKEVDEQWLRGRFGREALQWLRGRFGR
ELAEQWLRGRFGKEAEEQWLRGRFGKESEDEAEESQWLRGRFGREAEQWLRGRFGKEAADQWLRGRFGKEAAD
QWLRGRFGREADKQWLRGRFGKEVNGQWLRGRFGREANGQWLRGRFGREADKQWLRGRFGKEVDEQWLRGRFG
REAEQWLRGRFGREIEQWLRGRFGREESHEKMTRELEQWLRGRFGRDAADQWLRGRFGRGEKTSEALPKRTDA
KDSKTNEKKSDDRFESELNSAVKAAKSS*
