# Supplementary material for: Global Neuropeptide Annotations From the Genomes and Transcriptomes of Cubozoa, Scyphozoa, Staurozoa (Cnidaria: Medusozoa), and Octocorallia (Cnidaria: Anthozoa)
Source: Front Endocrinol (Lausanne). 2019 Dec 6;10:831. doi: 10.3389/fendo.2019.00831 (PMC6909153; doi:10.3389/fendo.2019.00831)
Supplement: Supplementary file 3 [file Data_Sheet_3.PDF]

Supplementary Fig. 3. Partial or complete amino acid sequences of the pQPPGVWamide preprohormones in scyphozoans. The sequences are highlighted as in Supplementary Fig. 1.

Nemopilema nomurai

> Nemopilema nomurai isolate NNO-Tongyong01 scaffold33_contig28

MKLITLGVVLTFSISTLLVAKAEDVDDASLEPAPPGVHGLLTREGDDFEEDENDALEHERRENQPPGVWGKRD
SQPPGVWGKRSKQPPGVWGKRTSKEENRLHRKKENQPPGIWRRGENQPPGIWRRGENQPPGIWRREANQPPGI
WRRGENQPPGIWRRGENQPPGIWRREANQPPGIWRREANQPPGIWRKRENQPPGVWGKRSKGGHHATIGSEIK
GRAQIKVAKKGLNEREAALIRTVKRLRDALAKE*

Rhopilema esculentum

>GEMS01058479.1 TSA: Rhopilema esculentum c63002_g1_i1 transcribed RNA sequence

MRLITLGIVVLLSMSNCLLTTAEDSNDASLEPVPPGPRSLFTKEGDDFEEDENDFLEHERRENQPPGVWGKRE
NQPPGVWGKRSNQPPGVWGKRVSKEENQPPGVWRKKENQPPGVWRKKENQPPGVWRRDNNQPPGVWRKKESQP
PGVWRKRENQPPGVWGKRSNKKHEENFSGVIDGRAMKEIEKDARGLNERKIALIRTVKRLREALAKEYK

Aurelia aurita

>GBRG01058423.1:c521-3 TSA: Aurelia aurita comp183113_c0_seq1 transcribed RNA sequence

MQVLILVFISTLCLSSSVRGDDDKEAQWRPMPPGALGDDRLTSNHEKIKNDALGDGRKKSKGKSDSLIEDSLN
EGAFDDEELSAQGKDEGISARENQPPGTWRRREIQPPGVWGKRSNQPPGTWRKRGSQPPGTWRKKENQPPGTW
RKKENQPPGTWRKRENQPPGTWGKRSN
