# Supplementary material for: Global Neuropeptide Annotations From the Genomes and Transcriptomes of Cubozoa, Scyphozoa, Staurozoa (Cnidaria: Medusozoa), and Octocorallia (Cnidaria: Anthozoa)
Source: Front Endocrinol (Lausanne). 2019 Dec 6;10:831. doi: 10.3389/fendo.2019.00831 (PMC6909153; doi:10.3389/fendo.2019.00831)
Supplement: Supplementary file 4 [file Data_Sheet_4.PDF]

Supplementary Fig. 4. Partial or complete amino acid sequences of the CNSPMCWFRGamide preprohormones in scyphozoans. The sequences are highlighted as in Supplementary Fig. 1.

Nemopilema nomurai

>GHAR01038137.1 TSA: Nemopilema nomurai nem_comp53321_c0_seq1, transcribed RNA sequence

MDVVWLMLFFITVTSTTYGRSTGKRATIDAEANELTIDERRQPFENDLDIDIYKAAFRSGKRECTSPMCWFRP
GKRNSRFNVETEKSKKRASSRVEESCNSPMCWFRGGKKRAVDFAEIAKRLLHRKPALKRFLQNVMHRQRKTQR
QVSEDNCDSPMCWFRPGREVIENAKKGKAKMSRGNWKSFFNKKIQRRIDTSMRGKSVMDENHLTNRKWKATRD
IGAGDSSSLLWEKDNAAY

Rhopilema esculentum

>GEMS01035705.1 TSA: Rhopilema esculentum c44618_g1_i1 transcribed RNA sequence

MGVIKLALVFVTLVSFANGRSTEKRTEIDAEANELTIDQRREPFENDLDIDIYKAAIRSGKRDCTSPMCWFRP
GKRGMLPRFTLGSSKRKSNDRNGESCNSPMCWFRAGKKRAFDFGEIAKRLLLRKPALKRFLHNVMHRHKKLQR
QVSEDNCDSPMCWFRPGRQVNDEKKKKEKRVINMNSGERYFKTQTQKRAGLTATGKKETSTKLSVGRGRNVDT
HGAAEDSDYQTWKKEYVVD

Aurelia aurita

>GBRG01085764.1 TSA: Aurelia aurita comp183113_c0_seq1 transcribed RNA
MHLRRTIFIVVFAFSVIKAAPLENRKKRCQSRNCNSETEEIHLDHIRNLSEDAIDIDLYEAFTTAVKRGCSSP
MCWFRPDGKRGMMLKRGLTRREKRKNHKRSSQSLKRVERKREEMVKQKKNCASPMCWFRATGKRREMMSQGQK
ELFVKRMALKRILKGMRDKRKREQEGETKTGCSSPMCWFRAGREMDNNSDKILQPDNEETKKQGYSEKHRRRD
PGEAAAKQIRMMKDAAERMQKKFVEEGKMFGPRDASMFGARGGRVLGAGEANVFRDEDDNDHLELSAA*
