# Supplementary material for: Global Neuropeptide Annotations From the Genomes and Transcriptomes of Cubozoa, Scyphozoa, Staurozoa (Cnidaria: Medusozoa), and Octocorallia (Cnidaria: Anthozoa)
Source: Front Endocrinol (Lausanne). 2019 Dec 6;10:831. doi: 10.3389/fendo.2019.00831 (PMC6909153; doi:10.3389/fendo.2019.00831)
Supplement: Supplementary file 5 [file Data_Sheet_5.PDF]

Supplementary Fig. 5. Partial amino acid sequences of the pQHLRYamide preprohormones in scyphozoans. The sequences are highlighted as in Supplementary Fig. 1.

Nemopilema nomurai

> Nemopilema nomurai isolate NNO-Tongyong01 scaffold11_contig2, whole genome shotgun sequence

MMSKTFPTTLILLMVGQFVGFCACKAVVNPGIFLLEADVPQTGQMPEGIGNKASYSELWEASVGSQDEPEEEG
IPHLRMGKETAPHMRYGKDFVDFLLHGAKNDAAPHLRYGKDVDENVRYGREVEVNIGYRGEMPPQLRYGKEVK
RQLRYGKEADQLRYGKEATQHVRYGKEAQHLRYGKEAQNLRYGKEAENVRYGKEAQHLRYGKEASNVRYGKEA
ANVRYGKEVDNVRYGKEAANVRYGKEVDNVRYGKEAANVRYGKEAANVRYGREAANVRYGKEVDNVRYGKEAS
NVRYGKEAANVRYGKEAQHLRYGKEAQHLRYGKETQHLRYGKEESIQHLRYGKEVGENSEQGKQGPVDAKLLI
GGHYEAARETDENGADVENAIWGTVIHSKTGEDILNVLKNLKALKQKPVSDCLTHLTTSIR

Rhopilema esculentum

>GEMS01049336.1 TSA: Rhopilema esculentum c56832_g1_i1 transcribed RNA

MTKSFPACLVFLIATEYIGLCCCKALVNPGVFLLEADMPFKEQISQEIETKPDPSNMWKPSEESDEEGYENGI
PHLRYGKEAAPHLRYGKELENFFHYGEESDDAQHVRYGKEADQHVRYGKEADDHVRYGKELQHLRYGKEAQHV
RYGKEAQHVRYGREAVQHVRYGKEVKKQLRYGKEAKHVRYGREVDQHVRYGKEAQHK

Aurelia aurita

>GBRG01040571.1:426-938 TSA: Aurelia aurita comp172589_c0_seq1 transcribed RNA sequence

MTQNALFLAIAVLCSNYLYQCGCRSIQDTDVFLLDADNPLRDDEQDSFDDIDLRELAPHLRYGKEVAPHVRYG
KEVAPHVRYGKEVAPHVRYGKEATQIDDIDRHQRYPARMPGKDFAVHGKLLSDLASGTQVDDKAEHGRRMSAL
MKILRDVASESTKLHRKRAVKTNA
