# Supplementary material for: Global Neuropeptide Annotations From the Genomes and Transcriptomes of Cubozoa, Scyphozoa, Staurozoa (Cnidaria: Medusozoa), and Octocorallia (Cnidaria: Anthozoa)
Source: Front Endocrinol (Lausanne). 2019 Dec 6;10:831. doi: 10.3389/fendo.2019.00831 (PMC6909153; doi:10.3389/fendo.2019.00831)
Supplement: Supplementary file 6 [file Data_Sheet_6.PDF]

**Supplementary Fig. 6.** Partial or complete amino acid sequences of the pQPLWSARFamide preprohormones in scyphozoans. The sequences are highlighted as in Supplementary Fig. 1.

**Nemopilema nomurai**

>Fgenesh_Nemopilema_nomurai_isolate_NNO-Tongyong01_scaffold18_contig1

MAFRTTLVLLIAMLLFTAEVASVATYLQGESSGGQQQHRPHSPGPSESKYPIMSSFFDKPQTQEGVGRQGSDK
LNGLTNPNGMGGFTSHEKPSSDYFLGKASSDYVQGNFYDGWARDIKTMNDGNEIQRVQLTNLDADASARPKRR
LQPLRYNDDLSWSSRYGLDTESQSSNPTIARVVTANNEQETSMDESGAKMEDIETNQLYRRNAKVDLNPPFWS
GRYGRAALDSNAEYSDSPPLWIGRFGKEAGATDDRQLVGKEAQKGNSQPLWTGRYGREVKDVNSQPLWSARFG
REVKDVNSQPLWSARFGREAVEGNDQPLWSARFGREVKDVNSQPLWSARFGREVKDVNSQPLWSARFGREVKD
VNSQPLWSARFGREAVEEVKDVNSQPLWSARFGREVKDVNSQPLWSARFGREVKDVNSQPLWAHGLEERSKML
TVNLCGAHGLEERSKMSTVNPCGAHGLEEVKDVNSQPLWSARFGREAVEGNDQPLWSARFGREVKDVNSQPLW
SARFGRRSKMLTVNLCGAHGLEERSKMLTVSLCGAHGLEERSKMSTVNPCGAHGLEERSKMLTVNLCGAHGLE
ERSKMSTVNPCGAHGLEERLWKVMTSPCGAHGLEERWKVTVNPCGAHGLEERSKMLTVNLVERTVGREVKDVN
SQPLWSARFGREVKDVNSQPLWSARFGREAVEGNDQPLWSARFGREAVEGNSQPLWSARFGREVKDVNSQPLW
SARFGREVKDVNSQPLWSARFGREVKDVNSQPLWSARFGREVKDVNSQPLWSARFGREVKDVNSQPLWSARFG
REVKDVNSQPLWSARFGREVKDVNSQPLWSARFGREVKDVNSQPLWSARFGREAVEGNDQPLWKARFGREVTN
VNSQPLWTGRYGREAYINADQKRVKKEAEEVNSQPLWSARFGKETNAIDEEGDPGKLVMHKRKKLTNGLEAED
TVQRPLWASRYGRDTTQKRKVRAGRRGTRVKDVTGRLIHRSSKGDTEKQEFQEDTDEDGGRGVGSEEEETPQH
LSGERFADDATGTRLLSSIERLRIALHRVSGSNEMKLGPLIKLLRGKFEVAEQKSKRTSARRRGKKLTWRKAT
SDELKRFDDILHSKIGLRESDTRQIQSKRRNAEAKTRAIKTDF

**Rhopilema esculentum**

> Rhopilema esculentum c12255_g1_i1 transcribed RNA sequence

MVRLCAMFLPLLTEILFTVNTASKLTRTGVYSYEVPQKDHPHKANSRHLSVLEYPQQLTSSNQHDDVEDLSSQ
NGKNLKDENGFRNFRIRSFEHLQRKRSLSKNRSGKEKIIKRREGTRGVQIKNFFIDPYGELDQKKGMTYQKKN
MLWNLRYGRETFSKMYQPLWNGRYGRKTYPTDEQWRQGRDTEEVNGQLRQRRETGE

> Rhopilema esculentum Unigene0033256 transcribed RNA sequence

QPLWSSRFGREANAVNEQLRPGREAEEANSQPLWSSRFGREANAVDEQLRPGREAEEANSQPLWNSRFGREAN
VVNGQLRQRRETGEVSSQPLWSSRFGREANAVNEQLRPGREAEEANSQPLWSSRFAGREANVLNGQLRQGRE

**Aurelia aurita**

>GBRG01065731.1:1025-1456 TSA: Aurelia aurita comp185179_c0_seq1 transcribed RNA sequence

MHWNARSCLLVCILNLARTDLLNVLASNHVHQGGVGNDEHIHRQGAGNFEPVYSDVGTRDTREDLNDKQEVRH
SADHSDTDSDEGGGPVIEPEEPVEEHRHRRSFINPGLLFSRYGRVAKAQGVEALPLHRKRMEYNARQRRELYA
DEVSTAGYEDVRDRMEHKRSTSNKFGKEAHEMKMPSSATRKRRNVVYSNNQHRTGARYDSEAENEKPFWKVRF
GREETIGPLWSARFGRELRDKGPLWKSRYGREGREVAPPWASRYGRDAQEKSLIMSSREAHDKSPFWNGRYGR
EEGHETPFWKGRYGREEGHETPFWNGRYGRESANEKPPWAYRIGRDVEESKSLVREAAPHMWKSRFGRDSKDR
REQVAKQKRMEAAINMELMAEPEGGHEQYLARHGKNGEEQELENEDTQPSVRFGRAVVSADAVDVDRDGSRMK
RGWKGRYGRVNPRIVNHIVVPQRSHGSGKEFMNYRPIMYAEQNVDGEVNTQRYDSKMDNWPARSKSQGVADLE

RQGGAGSERADAERDKTLEENDLAGTGFDGVDGVASTNDRQAEDRYGNRFLFQMERLRQKIRDTEMKEGLAAT
GEGTGFSRGKSDRGTSAGRPRSRNLNWEMENAND*
