# Supplementary material for: Global Neuropeptide Annotations From the Genomes and Transcriptomes of Cubozoa, Scyphozoa, Staurozoa (Cnidaria: Medusozoa), and Octocorallia (Cnidaria: Anthozoa)
Source: Front Endocrinol (Lausanne). 2019 Dec 6;10:831. doi: 10.3389/fendo.2019.00831 (PMC6909153; doi:10.3389/fendo.2019.00831)
Supplement: Supplementary file 7 [file Data_Sheet_7.PDF]

Supplementary Fig. 7. Partial amino acid sequences of the RPRSamide preprohormones in staurozoans. The sequences are highlighted as in Supplementary Fig. 1.

Calvadosia cruxmelitensis

>HAHC01040962.1 TSA: Calvadosia cruxmelitensis, contig Ccrux.16828.c0_g4_i2, transcribed RNA sequence

MVSAFTTGCKVYGLLLLLSINAACSPMRDQESTDEDDQSVWEWIQSYLNGDSYEGEAEESLRSIVEDSNQFKR
MKKASNEEIRPSADERDARNMLLASLLYDRYYEDLTENRDYSRSQENDHVTAAEYMNSLRERLVSNGLKAGKR
DDDMWLLTNRLHGAFRPRSGKRSEEEDRPRSGKRAEKDRPRSGKREDEDRPRSGKREDEVRPRSGKREYEDRP
RSGKRENEDRPRSGKREDEDRPRSGKREDEVRPRSGKREYEIRPRSGKREDEARPRSGKREDEDRPR

Haliclystus auricula

>HAHA01075613.1 TSA: Haliclystus auricula, contig TRINITY_DN11232_c0_g1_i3, transcribed RNA sequence

MKSFYVNAVCLCCILTFSHYAFSQPIDDWLSNYDSATDSVDEEEFKRTVARPRSGKRDEIEDLNDLLAAMNKN
EELYKELEEESVTEREAFRPRSGKRSKEGRPRSGKRDVEDRPRSGKRSELERPRSGKRETLRPRSGKRETLRP
RSGKRSEAERPRSGKRSEIERPRSGKRTEMDRPRSGKRETRPRSGKRSSFERPRSGKRSETERPRSGKRVEHS
RPRSGKRADLLNLIAEEINSMDRPRSGKRDVTDDINTNDDVNEVYTVDNDMWKSMKR

Haliclystus sanjuanensis

>HAHB01061752.1 TSA: Haliclystus sanjuanensis, contig Hsan.61752, transcribed RNA sequence

MKLFHVNAVFLCCTLTFTHFAFSQPIDDWLSTYDSVTDSANEEDFKRALDRPRSGKRDEIEDLNELLAAMTKN
EELYNELEEEPSEEGDEFRQRSGRSKDDRPRSGKRAADVRPRSGKRSEMERPRSGKREMSRPRSGKREFSRPR
SGKREALRPRSGKRSETERPRSGKRSEIERPRSGKRSETARPRSGKRETRPRSGKRSELERPRSGKRSEIERP
RSGKRAESSRPRSGKRAELLNLIAEELYAMDRPRSGKRAFDSRPRSGKRDLDDNTTYNDKFNELDTAADNDMW
SSMKRSTSDIFDSTTSSDTTNYDNEANNIEM

Craterolophus convolvulus

>HAGZ01021818.1 TSA: Craterolophus convolvulus, contig Convo_TRINITY_DN2329_c0_g1_i1, transcribed RNA sequence

MIIVLFVLPTLLTCAVIRDANGLDASLSSDNDHWDGIKTCLENALDDESTEEGPDAYNGEETWEGEDSEKKAA
FTWLMSKIMGEDGDESFEGEDEDEEIRFSENEGIFRAGDDRPRSGKRYTEEDRPRSGKREAENRPRSGKREAE
DRPRSGKREDRPRSGKREAENRPRSGKREAEDRPRSGKREDRPRSGKRE

Lucernaria quadricornis

>HAHD01049480.1 TSA: Lucernaria quadricornis, contig TRINITY_DN9917_c0_g1_i1, transcribed RNA sequence

MHRSVVAVVALVLLASSNGLPTRDADQGEGVGAMLKWERLYNYINSDTYSREDNSFTDYLRKLFPGNDDDEET
DEAGNEERRDAKKTSLSYRDYVAELSRSKTMQDNRDAAEELRECTDEGYSTQDDAEKRNINAVELSYRPRSGK
RKAEEERPRSGKRSEDDRPRSGKRSEDEKPRSGKRSEDDKPRSGKRSEDEKPRSGKRSEDDKPRSGKRSEDDK
PRSGKRSEDDKPRSGKRSED
