# Supplementary material for: Global Neuropeptide Annotations From the Genomes and Transcriptomes of Cubozoa, Scyphozoa, Staurozoa (Cnidaria: Medusozoa), and Octocorallia (Cnidaria: Anthozoa)
Source: Front Endocrinol (Lausanne). 2019 Dec 6;10:831. doi: 10.3389/fendo.2019.00831 (PMC6909153; doi:10.3389/fendo.2019.00831)
Supplement: Supplementary file 8 [file Data_Sheet_8.PDF]

Supplementary Fig. 8. Partial amino acid sequences of the GRFamide preprohormones in staurozoans. The sequences are highlighted as in Supplementary Fig. 1.

Calvadosia cruxmelitensis

>HAHC01044519.1 TSA: Calvadosia cruxmelitensis, contig Ccrux.17646.c0_g1_i1, transcribed RNA sequence

MNLLQYSAAILLLVSLAHCMPGHSKRDHVIEEIDEIDEVDDREMHSTHDENERRENHHDDEEKRGAHEPRATIEHTRGLDERREIHEHDTEHEKRNLGAREAKEQFLRGRFSREMEEQFLRGRFGREALSQFLKGRFGRESEDQFLRGRFGRESMEQFLKGRFGRESESQFLRGRFGREAMEQFLRGRFSRELEEQFLRGRFGREMEEQFLKGRFGREDMEQFLRGRFGRSSDEGSSNELREYFAASRYHNGDADSQAYEDKRSLQMKK

Haliclystus auricula

>HAHA01071884.1 TSA: Haliclystus auricula, contig TRINITY_DN13894_c0_g1_i2, transcribed RNA sequence

RGRFGREMEEQFLRGRFGREDMEQFLRGRFGRELEGQFLRGRFGRESEDQFLRGRFGREQFLRGRFGRETEDQFLRGRFGREDMAQFLRGRFGREVEEQFLRGRFGREDMEQFLRGRFGRENHGKESREFYAARIDDHDDYDTNEKRSTP

Haliclystus sanjuanensis

>HAHB01040451.1 TSA: Haliclystus sanjuanensis, contig Hsan.40451, transcribed RNA sequence

MKATCLLVLLCAIISIQCLPHNKRALEDHVVEEIDEIDEQDKRDAKRTSHESNTKREDDHHNEDRREQFLRGRFGREMEEQFLRGRFGREDMEQFLRGRFGRELEEQFLRGRFGREKEEQFLRGRFGREQFLRGRFGREMEGQFLRGRFGREDMAQFLRGRFGREMEEQFLRGRFGREDMEQFLRGRFGRENHGKESREFYAARIDDHDGYDADEKRSTQLK

Craterolophus convolvulus

>HAGZ01051122.1 TSA: Craterolophus convolvulus, contig Convo_TRINITY_DN6267_c0_g2_i1, transcribed RNA sequence

MNFVVLWMAIGLMSTAAHGLPAQDKRSAAEHVIEEIDEIDETDERESHPGGEEKRHSHEPRGMVEHTRSENERREHAHHDESHEERSSHPDREAREQFLRGRFGREGMSQFLRGRFGREDEEQFLRGRFGREDMEQFLRGRFGREAMHQFLRGRFGREDEEQFLRGRFGRELEAQFLRGRFGREAMHELLRETYGHNHDEEGKELREFVSARYDSGEADSRHYDDKRSLHGERK

Lucernaria quadricornis

>HAHD01021334.1 TSA: Lucernaria quadricornis, contig TRINITY_DN15109_c1_g3_i1, transcribed RNA sequence

MSDHVIEVDEIDEDIDRETERKEATDEPRAATEDERSMHEKRHTSDSERTTREAAEQFLRGRFGREAVAQFLRGRFGRETEEQFLRGRFGREAVAQFLRGRFGRETEEQ
