# Supplementary material for: Global Neuropeptide Annotations From the Genomes and Transcriptomes of Cubozoa, Scyphozoa, Staurozoa (Cnidaria: Medusozoa), and Octocorallia (Cnidaria: Anthozoa)
Source: Front Endocrinol (Lausanne). 2019 Dec 6;10:831. doi: 10.3389/fendo.2019.00831 (PMC6909153; doi:10.3389/fendo.2019.00831)
Supplement: Supplementary file 9 [file Data_Sheet_9.PDF]

Supplementary Fig. 9. Partial amino acid sequences of the pQPPGAWamide preprohormones in staurozoans. The sequences are highlighted as in Supplementary Fig. 1.

Calvadosia cruxmelitensis

>Ccr AWamide

MHVILSLLLVFIVNHMTEAGSHHHDNLGPMPPAIRKEIDEIYEDMISKELNEDLPDIYDKRENQPPGAWGKRENQPPGVWGKRENQPPGAWGKRENQPPGAWGKRENQPPGAWGKRENHQPGAWGKRENQPGAWGKRENQPPGVWGKRENQPPGVWGR

Haliclystus auricula

>HAHA01038762.1 TSA: Haliclystus auricula, contig TRINITY_DN7857_c0_g2_i1, transcribed RNA sequence

QPPGVWGKMENQPPGVWGKRENQPPGVWGKRENQPPGVWGKRENQPPGVWGKRENQPPGVWGKRENKRENQPPGVWGKRENQPPGVWGKRENQPPGVWGR

Haliclystus sanjuanensis

>HAHB01030546.1 TSA: Haliclystus sanjuanensis, contig Hsan.30546, transcribed RNA sequence

RENQPPGVWGKRDNQPPGVWGKRENQPPGVWGKRDNQPPGVWGKRENQPPGVWGKRDNQPPGVWGKRENQPPGVWGKRENKKENQPPGVWGKRENQPPGVWGKRENQPPGVWGKRENQPPGVWGKRENKRENQPPGVWGKRENQPPGVWGKRENKRENQPPGVWGKRENQPPGVWGR

Craterolophus convolvulus

>HAGZ01008087.1 TSA: Craterolophus convolvulus, contig Convo_TRINITY_DN5429_c0_g1_i1, transcribed RNA sequence

EIDDLYEEIFGKEEDKKVSDVLDRRENQPPGVWGKRENQPPGVWGKRENQPPGVWGKRENQPPGVWGKRENQPPGVWGKRENQPPGVWGKR

Lucernaria quadricornis

>HAHD01030305.1 TSA: Lucernaria quadricornis, contig TRINITY_DN14230_c0_g4_i1, transcribed RNA sequence

MSNCLLASLALVFLSHYIEAYEHAEDLSRPLPPSIRKEIDDLYDEVMRRTEESHYSAGEKRESQPGRSGKRENQLPTGTWGKREKIPTGVWGKRENQPPKGTWGKRQDIPTGVWGKRENQPPKGTWGKRQDIP
