# Supplementary material for: Global Neuropeptide Annotations From the Genomes and Transcriptomes of Cubozoa, Scyphozoa, Staurozoa (Cnidaria: Medusozoa), and Octocorallia (Cnidaria: Anthozoa)
Source: Front Endocrinol (Lausanne). 2019 Dec 6;10:831. doi: 10.3389/fendo.2019.00831 (PMC6909153; doi:10.3389/fendo.2019.00831)
Supplement: Supplementary file 10 [file Data_Sheet_10.PDF]

Supplementary Fig. 10. Partial amino acid sequences of the preprohormone for GPRGamide or related neuropeptides in Octocorallia. The sequences are highlighted as in Supplementary Fig. 1.

Renilla reniformis

>Renilla reniformis RGamide

GPRGGRTLTFDGEEGPRGGRTVTFDGEEGPRGGRTLNFDGKAGPRGGRTITFDGEAGPRGGRTLNFDGKAGPR
GGRSLELLDGDNLLPIDL*

Eleutherobia rubra

>gb|GHFI01021067.1|_translation_frame_+2

MKTLQGSGVVLLLSMQFWLAVKGNMNLNWEDYTGNAPQIEETPRAGKRTIDLYGIERPRGGKREIDLAGIEKP
RGGKREINLAGIERPRGGKREIDLAGI

Xenia sp.

>GHBC01017757.1 TSA: Xenia sp. KK-2018 xen_tr14519_c0_g1_i1, transcribed RNA sequence

MIQALTVLVLLVQCFCTIQAEFTSEENHRSQDLIESPRGGKRNFDFNEAPRGGKRSLDFNEAPRGGKRNFDFD
EAPRGGKRNFDFDEAPRGGKRNFDFDEAPRGGKRNFDFDEAPRGGK

Briareum asbestinum

>Briareum asbestinum sequence

MKTSAIVLLLVLKCWKVTNARRDTALSNGYNKRTIDFAGREEPRGGKRTIDFSGIEEPRGGRRTIDFAGREEP
RGGKRTINFAGREEPRGGKRTIDFAGIEEPRGGKRTIDFAGIEEPRGGKRT

Clavularia sp.

>GHAW01021700.1 TSA: Clavularia sp. cla_tr20062_c0_g2_i1, transcribed RNA

MKITTLRVVVFLSIQSLLAVKGREEDWIDTRYTPRVGERTINLAGIEAPRGGKRTIDFAGIEAPRGGKRTIDL
AGIEAPRGGKRTIDLAGIEAPRGGKRNIDLAGIEAPRGGKRNIDLAGIEAP

Heliopora coerulea

>SRA:SRR5949848.8505612.1 Heliopora coerulea

IEAPRGGKRTIELAGREAPRGGKRTIDL

Acanthogorgia aspera

>SRA:SRR3303654.3569131.1 Acanthogorgia aspera

SLDGIEKPRGGKREIDLDGTEKPRGGKREIDLD
