# Supplementary material for: Global Neuropeptide Annotations From the Genomes and Transcriptomes of Cubozoa, Scyphozoa, Staurozoa (Cnidaria: Medusozoa), and Octocorallia (Cnidaria: Anthozoa)
Source: Front Endocrinol (Lausanne). 2019 Dec 6;10:831. doi: 10.3389/fendo.2019.00831 (PMC6909153; doi:10.3389/fendo.2019.00831)
Supplement: Supplementary file 11 [file Data_Sheet_11.PDF]

Supplementary Fig. 11. Partial amino acid sequences of the Antho-RFamide (pQGRFamide) preprohormones in Octocorallia. The sequences are highlighted as in Supplementary Fig. 1.

Renilla reniformis

>Renilla reniformis RFamide

MDLPCYFTVLLLILNTHTLAAPSTSEGLDERNLLDKTELSINDEIFSEDDDVLARDAEDKQGRFSRKLNNKLN
DAVQGRFGRNKKEEQGRFGRENKEQGRFGRENEEQGRFGRESEEQGRFG...

Eleutherobia rubra

>EruRFamide

MSYMFVLLLLCSVLSSIAPAAVDKVQKDESMALNTELTRKEVSHEAAELVLNDKSSGADGHEMARELNTDQGR
FGRNGLLNQGRFGRAEETQGRFGRESKAKKDQGRFGRESKSDQGRFGRESKTDQGRFGRESKMDQGRFGRESK
TDQGRFGRESKSDQGRFGREFKTDQGRFGRESKTDQGRFGRESKTDQGRFGRESKTNEENEKKSTQGRFSRGN
MDDDVFELQGRFGRKYENQGRFGRKYSKDDQGRFGREVAKADEQGRFGRFLENNEFSESQGRFGRDFLRNEEQ
GRFGRSEDQGRFGRELWKNDDQGRFGREFSETKEQGRFGREDFMNNEQGRFGRMVRDLLKDQGRFGRNFLRNE
EQGRFGREISESDEQGRFGREDKNNEQGRFGRELLKDQGRFGKK

Xenia sp.

>gb|GHBC01044262.1|_translation_frame_+3 Xenia

MNTLTLLLVSSILSSAFPLNAVEEVHRNDLIETARGPGFDESLKRAMHFEQGRFGRENSVMFDLNQGRFGREK
QGRFGRETQGRFGRTNVADISAEVQGRFGREDKQGRFGRKVDSQGRFGRESTKKFENERFARVQVGRDEQGRF
GREFLNSQGRFGKK

Briareum asbestinum

>GHBD01017447.1 TSA: Briareum asbestinum isolate digitate
B_asbestinum_21019, transcribed RNA sequence

MKSALFVLSIFLFNAVTIAVSSDETIALGAEINQEDIDKDDTEVLFSDIKDKTETKKSIEADKSNMNNEEVAV
NDNEDSQGRFGRDDMVKQGRFGRTQGRFGKDLENMNDNKVELDLSFARNLDQDFSTNQGRFGRKFSSADMKNN
QGRFGREHLDLIEQGRFGRENEDQGRFGRENEDQGRFGRDNEYQGRFGRENEDHGRFGRESKDQGRFGREKND
QGRFGRENKDQGRFGRDSVESNEQGRFGRNKLDAEEQGRFGRDFSKFGTQERFGREFLESEDQGRFGREFSEN
EEQGRFGRRYSTENSNNQ

Clavularia sp.

>GHAW01192256.1 TSA: Clavularia sp. cla_tr102569_c0_g1_i1, transcribed
RNA sequence

MDLLPALILACSLLAVISLGAVDAVETVNRNAYQGRFGRESEIDGGKENARVASDSMDLDQGRFGDVDVFSNQ
GRFGRETKDNSDVDGEDDTDNEVYEVQGRFGRKFVDQGRFGRKLRDYQGRFGRVESSESNEQGRFGRKFLENE
ELGEMKDQGRFGREVVKNEDQGRFGRNMADFTQGRFGRKLEVGEEKGLSRDIVENDNQGRFGRGFVNDEKQGR
FGRKLVDKKDQGRFGREEHGRIFLKNENDEQGRFGRNFRSNDASKTQGRFGK

Heliopora coerulea

>gi|1209670625|dbj|IABP01006161.1|_translation_frame_+3 Heliopora

MNYVLILLLCFLLSAVSQAANTATSHDKTLALDAGLIREDVDKDNTELLFNQEDIDKAAEKSLQEVETRANNE
ELAARNVEESQGRFNRDSIAGQGRFGREMDQGRFGRDSVESFTNQGRFGRMFWKSNNIEDQGQFGRNYGNSVE
QGRFGRENEEKDEQGRFGREFSKKSEQGRFGRDFSDHKDQGKFERDFSENKDQGRFGRDFSENQEQGRFGKDF
SEKDANGKLKQEFSDNGEQGRFGRGFSAEDSIVQGRFGRESAAQGRFGRFVRKNEMTSEQGRFGRKM

Acanthogorgia aspera

>gb|GETB01014884.1| TSA: Acanthogorgia aspera T2_Unigene_BMK.15056
transcribed RNA sequence

MSSMLALLLVLSILSSVTPTAVNEVQNDEAVAVSEEVVNGKDVTEAFFNDKENEVQRDEDSNVEQDETKASEV
NSVQGRFGRSGILNQGRFGRTEETQGRFGRESDQGRFGREETHSDGKRENNQGRFGRESMNQGRFGREKANGD
EKSSEQGRFGRESMDQGRFGRDNVHIRAGIDMSNFDQGRFGRMVLERFAREKQGRFGRDENAIQGRFGREETD
NDNFEIQGRFGRKLSKQGRFGRDFDKKTEQGRFGRKISAKDVSEDEEQGRFGRKLLEFDNQGRFGREFSENVE
QGRFGREFSENDEQGRFGREFSETKQGRFGREFSEREQGRFGRGYEKQGRFGRESSDNDEQGRFGRELAESEV
QGRFGRDYSEENEQGRFGRGFEKNIDQGRFGREFSKTKEQGRFGRTSMFQGRFGRAHHY
