# Supplementary material for: Global Neuropeptide Annotations From the Genomes and Transcriptomes of Cubozoa, Scyphozoa, Staurozoa (Cnidaria: Medusozoa), and Octocorallia (Cnidaria: Anthozoa)
Source: Front Endocrinol (Lausanne). 2019 Dec 6;10:831. doi: 10.3389/fendo.2019.00831 (PMC6909153; doi:10.3389/fendo.2019.00831)
Supplement: Supplementary file 12 [file Data_Sheet_12.PDF]

Supplementary Fig. 12. Partial amino acid sequences of the pQLRGamide or pQLRAamide preprohormones from seven Octocorallia species. The sequences are highlighted as in Supplementary Fig. 1.

Renilla reniformis

>FXAL01074665.1 Renilla reniformis genome assembly, contig: Renilla_reniformis.v1.074665, whole genome shotgun sequence

MRSAILFITTLYLCSISASHVKKPSDDQLRGGRDTIPINQYGRSTIPKEQLRGGRDKIPEEQLRGGRDNIPDE
QLRGGRDSIPKDQLRGGRDTIPKEQLRGGRDSIPKEQLRGGRDAI...

Eleutherobia rubra

>GHFI01002883.1 TSA: Eleutherobia rubra isolate pohang1 contig2883, transcribed RNA sequence

MKTSLLLVLILCLWCVESTQLRGGRDKIPSVKTLDQLRGGRSKIANEKLHGGREYIPLNQLRGGRDAIAKEQL
RGGREIVLTDQLRGGRNIISGDQLRETIISSRQLRGGRNSIPNKQLRGGRNIISNDQLRGGRDVITNDQLRGG
RNTIVTEQLRGGRNVILHDQLRGGRNKIPSVQLRGGRDTIPDRQLRGGREVKLENQLRGGRDIITNEQLRGGR
EMKSEDQLRGGREMKSKDQLRGGREMKSKDQLRGGR

Xenia sp.

>GHBC01038083.1 TSA: Xenia sp. KK-2018 xen_tr26825_c0_g2_i1, transcribed RNA sequence

MKMKKFLYVVLIVLNLGLVECFPKKNRAGGDFEFVNQLRAGREIKPDYQLRAGREMKSDEQLRAGREMKSDEQ
LRAGREMKSDEQLRAGREMKSDELQRDGRETKSDEQLR

Briareum asbestinum

>SRA:SRR7895344.160770368.2 Briareum asbestinum
RSGRNTIPNNQLRSGRNVISDDQLRSGRAIILKDQLRNGRNSILYDQL

Clavularia sp.

>gb|GHAW01085241.1|_selection_translation_frame_+2 Clavularia

METSYLFILAACLMYCQASQVPAGRDEIPNKFISNDQLRAGRDIMADSQIRADKDNIPRHQLRAGRDAIFNDQ
LRAGRDNVPNNQLRAGRDTTVNDQLRAGRDNVPNNQLRAGRDTTVNDQLRAGRDNVP

Heliopora coerulea

>GFVH01060769.1 TSA: Heliopora coerulea Hcoe_TRINITY_DN22826_c0_g1_i1 transcribed RNA sequence

MKTVLLLAMASYLWCANGIKFNKIPKYELRSVRELISKDQLRGGRNAIPDAHGQLDEKRDFIANDQLRGGRNN
IAKDQLRGGRNTIPSSQLRGGRDVILRDQLRGGRNVIPKNQLRGGRDTIPGDQLRGGRDTIPSDQLRGGRDTI
TMDQLRGGRDTITSDQLRGGRDTIPSDQLRGGRDTITSDQLRGGRDTIPNDQLRGERNTIPSDQLRGGRDAVP
GDQLRGGRDEIPSNQLHGGQDIIPSDQFRPGQNTILSDQLRGGRDSITGDQLRGGRTIIPKDHMAAENNIRDR

Acanthogorgia aspera

>gb|GETB01013517.1| TSA: Acanthogorgia aspera T2_Unigene_BMK.13663 transcribed RNA sequence

MKISLLLTLAACLWCVESTQLRGGREMNDQLPGGRSFLSYDQLRGGRSKIEGEQLRGGRSKITSSQLRGGRDA
ITNDQLRGGRELTISAQLRGGRNTILHNQLRGGRDKILSDQLRGGRETIPNDQLRGGRNKIFKDQLRGGRNTI
ASDEQLRGGRNEIPNNQLRGGREVFSNQLRGGRELESKDQLRGGRDTIVDDQLRGGRGIITLDQLRGGREIIA
KSQLRGGREIIAAEDQLRGGREVIAKDQLRGGREITKKDQLRGGREIIAKDQLRGGRDVIAKDQLRGGREITS
KNQLRGGREIIAEDQIRPGREVSVESQLRGGREITAKDQLRGGREVIANDQLRGGRDTISKINKDVKN
