# Supplementary material for: Global Neuropeptide Annotations From the Genomes and Transcriptomes of Cubozoa, Scyphozoa, Staurozoa (Cnidaria: Medusozoa), and Octocorallia (Cnidaria: Anthozoa)
Source: Front Endocrinol (Lausanne). 2019 Dec 6;10:831. doi: 10.3389/fendo.2019.00831 (PMC6909153; doi:10.3389/fendo.2019.00831)
Supplement: Supplementary file 13 [file Data_Sheet_13.PDF]

Supplementary Fig. 13. Partial amino acid sequences of the pQPFHamide or PPFHamide preprohormones from six Octocorallia species. The sequences are highlighted as in Supplementary Fig. 1.

Renilla reniformis

>FXAL01052020.1_selection_selection_translation_frame_+2 Renilla reniformis

MSASSVLFALLCVALAATQASTADVDSSSLVENEDIEIFWDMDQRREESPVFHRRREELPPFHGKREEVPPFH
GKREEVPPFHGRREELPPFHG

Eleutherobia rubra

>Eleutherobia rubra isolate pohang1 contig3558

MHLETYKFVSFLLCILSITKDVYGASLLNDEQPFEIDYGKRKQPFHDKRGELEQPFHGKRELIDEQPFHDKRD
EFEQPFHGRRELTDEQPFHGKRDEQPFHG

Xenia sp.

>GHBC01034490.1 TSA: Xenia sp. KK-2018 xen_tr25091_c0_g9_i1, transcribed RNA sequence

EQPFHGKREFAGEQPFHGKREFSEEQPFNGKREFAGEQPFHGKREFAGEQPFHGKREFSEEQPFNGKREFAGE
QPFHGKREFAGEQPFHGKREFSEEQPFHGK

Clavularia sp.

>GHAW01115473.1 TSA: Clavularia sp. cla_tr75822_c0_g7_i1, transcribed RNA sequence

MAVTAERHFILVCVALALHGTCAGPLGNSVKVVTDGTELFVEGEFGEEKLHDSLKRELGNRRHLRKTDVHSRE
EQPFHGKREIAVKQPFHGRREMNKEQPFHGRREMNKEQPFHGRREMNKEQPFHGRRELVVDQPFHGRREMNKE
QP

Heliopora coerulea

>gb|GFVH01084024.1|_translation_frame_+2 Heliopora

FLDYEDVSKRARPFLGKRADETDGTEEETDFDKRARPFLGKRAYADDNDEKRARPFLGKRGDEEEAFEMMLPS
YYEESSEDKRARPFWGKRTLMEEEEKRARPFLGKRADDEDFEDKRARPFLGKREYDMEDDKRARPFLGKRSD
MEEEEKRARPFLGKRSDEEEKRARPFLGKRNMNDLDLDDEDIKRARPFLGKRSDEDNKRARPFLGKRDDFKFE
LDEEDNK
