# Supplementary material for: Global Neuropeptide Annotations From the Genomes and Transcriptomes of Cubozoa, Scyphozoa, Staurozoa (Cnidaria: Medusozoa), and Octocorallia (Cnidaria: Anthozoa)
Source: Front Endocrinol (Lausanne). 2019 Dec 6;10:831. doi: 10.3389/fendo.2019.00831 (PMC6909153; doi:10.3389/fendo.2019.00831)
Supplement: Supplementary file 14 [file Data_Sheet_14.PDF]

Supplementary Fig. 14. Partial amino acid sequences of the GPRRamide preprohormones from seven Octocorallia species. The sequences are highlighted as in Supplementary Fig. 1.

Renilla reniformis

>Renilla reniformis Sequence

MANICVLLVTLFFVAFAQSRSIQNTKNDELVEGLKNAFDETENIEENISVDNLPTRKGRDEITGPRRGRDEIT
GPRRGRDGITGPRKGRDEIT

Eleutherobia rubra

>GHFI01020780.1 TSA: Eleutherobia rubra isolate pohang1 contig20780, transcribed RNA sequence

MASFYCIVLVAISCVFLVECRHTASRDEEDFFKAFGNLYSDFEGPRFGDEMVELEELGPRRGREITLGPRRGR
DEIIEVDDDDEDNTSGPRRGRDKITGPRRGRDEITGPRRGRDEITGPRRGRDEITGPRRGRDEITGPRRGRDE
ITGPR

Xenia sp.

>GHBC01121041.1 TSA: Xenia sp. KK-2018 xen_tr53608_c0_g1_i1, transcribed RNA sequence

MAISVRLILALVCCTTIEFQSLTALEDRKELLKAFEDNDDEWRTPRLGDEIIELKEIGPRRGREYLLGPRRGR
DEINQDYFDIGPRRGRDEISAPRLGRDEITGPRRGRDEITGPRRGRDENSGPRRGRDKISGPRRGRNEISGPR
RGRDEITGPRKRRDETVGPRRGRDEISAPRLGRDEITGPRRGRDEITGPRRGRDENSGPRRGRDKISGPRRGR
NEISGPRRGRDEITGPRKRRDETVGPRRGRDE

Briareum asbestinum

>Briareum asbestinum sequence

RDEITGPRRGRDEITGPRRGRDEITGPRRGRDEISGPRRGRDKISGPRRGRDEITGPRRGRDEIVGPRRGRSE
IAGPRFGRDEIRGPRRGRNLATGPRRGRDDIQGPRRGRDEITGPRRGRNEISGPRFGRDEVIGPRRGRDEITG
PRRGRDEITGPRRGRDEITGPRRGRDEITGPRRGRDEINGPRRGREEIIGPRRGRNVITGPRRGRDLITGPRR
GRDDILGPRRGRDIVREQRVWRDEITGPRRGREEISGLRRTSETAGPRLGRNVYSAEDLESLAREFYEHNDSD
SLDDIYNDIDDYGDDTDDGNDYGNIDEGSNEMSARSNIIGHRRRNSDDFEFAVGDVDFSQFTELKK

Clavularia sp.

>GHAW01013696.1 TSA: Clavularia sp. cla_tr12713_c0_g1_i1, transcribed RNA sequence

MATLHIIFCLTISCMCLAECRHIQTSEENEWLEAFANVYNDLEGPRLGDEEYFNNYENEITKELMDSQRGRDE
ITGPRRGRDEITGPRRGRDEITGPRRGRDEITGPRRGRDEITGPRR

Heliopora coerulea

>GFVH01014783.1 TSA: Heliopora coerulea Hcoe_TRINITY_DN144511_c0_g1_i1 transcribed RNA sequence

DEIFGPRRGRDEIYGPRRGRDEISGPRRGREVKMGPRRGRSDITKGSSDGPRRGREVNSRQRRSIDLYHVAPRLGRNVDFGYSLDDEKHREMRKLQSLDENNFRGRYYDVNDDGDGYEDSVGSFNGESDNEMFLRSNMIGQRRRNYDNFEFEVGDFDFNGID

Acanthogorgia aspera

>GETB01029581.1_translation_frame_+1 Acanthogorgia aspera

MGTFHKIIFVAISCIFLAECRHIASHEENDLFDAFANVYNDFEKPRLGDERVVELEEIAGPRRGRQLIVETGEELDDDDEEEIAGPRRGRDEITGPRRGRDEITGPRRGRDEIT...
